# Biobanking knowledge and donation willingness among musculoskeletal patients in England: a multisite cross-sectional study

**DOI:** 10.1136/bmjopen-2025-111653

**Published:** 2026-02-15

**Authors:** Tracy Boakye Serebour, Angeliki Kerasidou, Naomi Gray, Xavier Luke Griffin, Chris Peach, Harvinder Pal Singh, Kim Wheway, Maria da Graca Ambrosio, Mathew Baldwin, Sarah Jane Bothwell Snelling

**Affiliations:** 1 Nuffield Department of Orthopaedics, Rheumatology and Musculoskeletal Sciences, University of Oxford, Oxford, UK; 2 University of Oxford Nuffield Department of Population Health, Oxford, UK; 3 Department of Paediatrics, University of Oxford, Oxford, England, UK; 4 Trauma and Orthopaedic Surgery, Queen Mary University of London, Whitechapel, UK; 5 Manchester University NHS Foundation Trust, Manchester, England, UK; 6 Faculty of Science and Engineering, The University of Manchester, Manchester, England, UK; 7 Leicester General Hospital, Leicester, UK; 8 University of Leicester, Leicester, UK; 9 University of Oxford Department of Social Policy and Intervention, Oxford, England, UK

**Keywords:** Orthopedics, Surveys and Questionnaires, Attitude, Genomic Medicine, Community-Based Participatory Research

## Abstract

**Abstract:**

**Objective:**

To date, few studies have investigated the factors associated with musculoskeletal patients’ willingness to donate biological samples and their knowledge regarding the use of such samples. We investigated the associations between these distinct knowledge factors, patients’ willingness to donate and socio-demographic factors.

**Design:**

Cross-sectional survey.

**Setting:**

Musculoskeletal outpatient clinics across four sites in England, representing varied demographic populations.

**Participants:**

A total of 469 adult patients attending musculoskeletal appointments were recruited through convenience sampling.

**Primary and secondary outcome measures:**

Ordinal regression models were employed to identify socio-demographic and clinical predictors of patients’ willingness to donate biological samples. Other outcome measures specifically in two areas of patient knowledge include: (1) knowledge of sample use and (2) knowledge of surgical waste tissue value and management.

**Results:**

Only 37% of participants were aware of the term ‘biobank’. Despite this, participants showed a high level of knowledge regarding both biological sample use and surgical waste tissue management, although their understanding varied considerably by ethnicity and education. Participants with no formal education exhibited a lower level of knowledge in both areas related to sample use and surgical waste tissue management for biomedical research ((OR 0.30, 95% CI 0.14 to 0.61; p=0.001); (OR=0.29, 95% CI 0.16 to 0.52, p<0.001), respectively). Individuals with ≥2 A-levels or equivalent (OR=0.44, 95% CI 0.24 to 0.79, p=0.006) and those identifying as Asian/Asian British (OR=0.51, 95% CI 0.29 to 0.88, p=0.016) also demonstrated a lower understanding of the value and management of surgical waste tissue. Willingness to donate was generally high but was significantly lower among non-Caucasian participants (Black (OR=0.23, 95% CI 0.08 to 0.61, p=0.004); Asian (OR=0.29, 95% CI 0.15 to 0.56, p<0.001)) and those with lower education levels (no formal education (OR=0.40, 95% CI 0.18 to 0.91, p=0.029); preferred not to disclose (OR=0.27, 95% CI 0.13 to 0.57, p<0.001)). Participants from Manchester were 2.18 times more likely to report a higher willingness (OR=2.18, 95% CI 1.09 to 4.33, p=0.027). Finally, participants who were aware of the term “biobank” had 2.16 times the odds of willingness to donate to biobanking initiatives for biomedical research (OR=2.16, 95% CI 1.23 to 3.77, p=0.007).

**Conclusions:**

Despite low awareness, musculoskeletal patients showed a high willingness to participate in biobanking. However, significant disparities by ethnicity and education persist. Targeted, inclusive engagement strategies are needed to address under-representation and foster informed, equitable participation of musculoskeletal patients in biomedical research.

**STRENGTHS AND LIMITATIONS OF THIS STUDY**

- This was a multisite cross-sectional study conducted across four National Health Service trusts in England.
- Ordinal regression models were used to identify predictors of knowledge and willingness, and missing data were handled via multiple imputation by chained equations.
- This study would have benefited from a mixed-methods approach that combined quantitative and qualitative data collection, providing deeper insight into participants’ reluctance to donate biological samples.
- Patient self-reported survey data may be subject to measurement and reporting bias.

## Introduction

 Musculoskeletal (MSK) diseases affected approximately 494 million people in 2020, representing the second leading cause of non-fatal disability worldwide.^1^ In the UK, over one-third of the population lives with an MSK condition, and projections indicate that these figures will rise significantly by 2050.^2^,^4^ Addressing this growing public burden requires sustained research efforts and the development of novel diagnostic and therapeutic approaches.

Recent developments in RNA sequencing methods, including single-cell and spatial multi-omics technologies, enable the study of cellular and molecular mechanisms in health and disease.^4^ However, the applications of these technologies rely on the procurement and/or biobanking of diverse human biological samples to construct single-cell MSK reference atlases.^5^

With the dynamic development of biobanks over the past few decades, well-established biobanking initiatives, such as the UK Biobank,^6^ provide critical infrastructure for collecting a vast array of comprehensive health-related data, including biological samples, genetic data and health data.^7^ However, their utility depends on public trust,^8 9^ engagement^10^ and willingness to participate.^11^ Understanding the population’s awareness and knowledge levels, as well as the factors influencing their willingness to donate, is especially relevant given the pressing demand for human biological samples (HBSs) to explore genetic variations and disease mechanisms.

Current literature indicates that knowledge of biobanks, public trust in research institutions and the government, as well as individual and collective beliefs, sociocultural narratives and religious beliefs, may influence public attitudes towards biobanks and their willingness to participate in biobanking initiatives.^12 13^ Several studies conducted in the UK have examined barriers to biobanking efforts, highlighting potential gaps and issues that need to be addressed to ensure the ongoing availability of health-related data and biological samples for biomedical research.^14^,^18^ The public generally viewed research involving donated HBSs positively, with most considering it important.^14 17^ However, concerns about commercial entities accessing biological data are more prominent than those about national academic institutions.^17^ Moreover, a general mistrust of researchers and their intentions is particularly common among ethnic minorities.^19^

Studies also indicate a moderate to high willingness among the public to engage in biobanking initiatives, though this varies by the type of sample requested.^17 18^ The public is more likely to donate ‘waste’ or diseased tissue, whereas certain tissue types, such as bone, eye and brain tissue, are considered more controversial^17^. Knowledge of biobanks was infinitesimal among the UK public, with an inadequate understanding of the use of HBSs (such as blood and urine) for biomedical research and very low knowledge of the use of surgical waste for medical research. These studies suggested that ongoing public outreach and education may address sensitivities toward certain sample types and improve public understanding of biobanking initiatives.

This study was conducted to advance our understanding of the factors influencing patient engagement with biobanking, particularly within the context of MSK research. It contributes to the Ancestrally Inclusive Musculoskeletal Single-cell Network,^20^ an international initiative that aims to develop the first ancestrally inclusive cellular atlas of the healthy human MSK system as part of the Human Cell Atlas project.^21^ The findings are intended to inform strategies to improve patient participation in donating HBSs for biomedical research in the UK and to complement other community engagement work at our international partner sites. Likewise, the findings obtained will support the development of patient-centred MSK research infrastructure and contribute to broader guidance on biobank engagement.

The study aimed to: (1) identify the factorsassociated with patient knowledge of HBS use and surgical waste tissue value and management; (2) examine the drivers of willingness to donate HBSs for research; and (3) assess whether awareness of key terminology related to biobanking is associated with willingness to donate.

## Methodology

### Study design

This study employed a survey-based, cross-sectional design. This approach was chosen to explore the influence of nine predictors, such as gender, age, ethnicity, region, history of past illness in the last 6 months, patient chief complaint, religion, native language, education and employment status, on patients’ awareness and knowledge of biobanking as well as their willingness to donate samples for biomedical research.

### Sample size determination

The required sample size was determined using Cochran’s formula for cross-sectional studies (n=*Z*^2^*p*(1–*p*)/*d^2^*). Assuming a conservative prevalence (*p*) of 50% to maximise variance, a 95% confidence level (*Z*=1.96) and a 5% margin of error (d=0.05), the minimum required sample size was calculated as 385. To account for potential non-response (10%), an inflation factor (1/(1–0.10)) was applied, resulting in a final target sample size of 428 participants. The final sample size of 469 participants exceeded this threshold, providing sufficient statistical power to meet the study objectives.

### Survey instrument

We developed a 27-item questionnaire based on previous studies^17 22^ on public attitudes towards donating biological samples for research. While these studies have investigated public knowledge and willingness to donate HBSs, we found no existing questionnaires that specifically assessed knowledge and awarness of biobanking among MSK patients across different population groups in England. This study population is particularly relevant to efforts to build ancestrally diverse MSK single-cell atlases^5^. Therefore, the survey was tailored to this specific population.

The survey consisted of four sections, including both closed-ended and open-ended questions.^23^ The sections covered: socio-demographics and health history (11 items), knowledge of HSBs for biobanking (9 items), willingness to donate (6 items) and awareness of the term ‘Biobanks’ (1 item). To identify reasons for unwillingness or hesitancy, participants could select from a list of common reasons identified in the literature^11^ or provide a free-text response. The survey did not include an option for participants to indicate they did not wish to respond to the items being queried.

### Instrument validity and reliability

Bartlett’s test of sphericity^24^ was significant, χ²(120)=3785.65, p<0.001, indicating that the correlation matrix was not an identity matrix and therefore suitable for factor analysis. The Kaiser-Meyer-Olkin^25^ measure of sampling adequacy was 0.86, exceeding the recommended threshold of 0.60, with all individual item values ranging from 0.79 to 0.92. These results show that the data were appropriate for exploratory factor analysis (EFA). In both analyses using imputed and nonimputed data, parallel analysis indicated a three-factor loading, confirming the survey instrument’s hypothesised three-factor structure (online supplemental figure 1).

The final EFA model identified a stable and clear three-factor structure: (1) ‘Willingness to donate’, (2) ‘Knowledge of HBS Use’ and (3) ‘Knowledge of Surgical Waste Tissue Value and Management’ (online supplemental table 3). However, two items assessing (a) knowledge of the terms ‘biological sample’ and ‘biospecimen’, (b) knowledge of patient health data being valuable for medical research, were removed from the final scales due to poor factor loadings, indicating they did not adequately reflect their intended constructs (online supplemental table 4). They were kept as individual items of investigation due to their relevance to the established literature regarding willingness to donate.^11^ All three construct scales demonstrated high internal consistency, with pooled Cronbach’s alpha values of 0.90, 0.84 and 0.78, respectively. The omega coefficients were also as follows, respectively: 0.91, 0.86 and 0.75 (online supplemental table 5) and supported a three-factor structure with an overarching general factor**,** with strong loadings for Factors 1–2 and comparatively weaker loadings for Factor 3 (online supplemental figure 2)

#### Data collection and sources

Data were collected between February 2023 and July 2024. A convenience sampling strategy was employed through recruitment at four clinical sites providing MSK outpatient services across four UK cities: London, Leicester, Oxford and Manchester. Patients recruited via this method in clinical settings may be more engaged with healthcare services, which could influence baseline trust or willingness to take part in research. Eligibility criteria included participants aged 15 or older with a MSK condition who were able to provide informed consent. Patients who lacked the capacity to consent or were unable to read English were excluded. Clinical research networks facilitated the recruitment of eligible patients. After providing written informed consent, participants completed a paper-based questionnaire. To increase the ethnic diversity of the sample, recruitment in Manchester and Leicester was prioritised to better reflect the clinical population. Of the 500 individuals who gave consent, 469 completed the survey (response rate 94%).

While complete elimination of all biases is not possible in observational studies, several steps were taken to minimise potential sources of bias. The use of multiple recruitment sites and oversampling from more diverse regions ensures broader representation. Questionnaire items were piloted with patient representatives to provide clarity and reduce measurement bias. Feedback was received on the survey’s comprehensibility, particularly regarding its ease of understanding for non-native English speakers. Data were collected anonymously to mitigate social desirability bias, and standardised procedures were used for data entry and transformation to reduce information bias.

### Statistical analysis

All analyses were performed using R (V.4.4.1). Quantitative variables are presented as absolute values, percentages or means with SD, as appropriate. The frequency of socio-demographic and clinical variables (both categorical and continuous) is reported without imputation.

Tests for missingness showed that data (0.4–9.2%) were not missing completely at random (MCAR) (Little’s MCAR test, p<0.05), with missingness significantly associated with age, employment status and religion (online supplemental table 1, 2). Given this pattern, multiple imputation by chained equations was employed to handle incomplete data, yielding five imputed datasets, each with five iterations. Following Beesley and Taylor’s method,^26^ the imputed datasets were stacked and analysed using regression models. Sensitivity analyses comparing complete-case data (n=465) and imputed data (n=2130) yielded generally consistent results. However, age was statistically significant in the complete-case analysis for knowledge of HBS use in biomedical research but not in the imputed dataset, supporting the decision to impute the data.

A cumulative logit model was used to determine the effect of predictors on knowledge outcomes and willingness to donate. Composite scores for knowledge and willingness were converted into ordered outcome categories (‘Low’, ‘Moderate’, ‘High’). Responses were scored as yes (3), not sure (2) and no (1) and summed across items. For ‘Knowledge of HBS Use’ and ‘Knowledge of Surgical Waste Tissue Value and Management’ outcomes, summed scores were categorised as (low (0–3), moderate (4–8) and high (9–12)), (low (0–3), moderate (4–6) and high (7–9)), respectively, while ‘Willingness to donate’ scores were categorised as low (0–6), moderate (7–12) and high (13–18). With ‘High’ as the reference level for the three constructs, regression models were fitted and used to estimate associations between predictors and outcome categories. Effect sizes were expressed as ORs with corresponding 95% CIs. Model fit and proportional odds assumptions were checked using the Brant test. No subgroup or interaction analyses were conducted because they were not specified in the study aims.

To account for the convenience sampling strategy and regional oversampling, all multivariable regression models included recruitment region as a predictor. This approach adjusts for potential confounding by regional variation in participant characteristics.

Statistical significance was defined as p<0.05. All statistical interpretations were considered in light of the cross-sectional design and data limitations.

### Patient and public involvement

Patients were initially involved during the study design phase through collaboration with the OPENARMS patient and public involvement group. Members of this group provided input that shaped the framing of research questions and helped develop survey content to reflect patient priorities. A multidisciplinary team finalised the survey and pilot-tested it with patient partners to ensure clarity, comprehensibility and relevance. Although the outcome measures were based on the literature and project objectives, patient feedback influenced the emphasis on specific themes, such as articulating common reasons for reluctance to donate and simplifying biobanking terminology for the questionnaire. Patients were not involved in recruitment or data analysis, but they were consulted in planning dissemination strategies, including lay summaries for participants and community outreach materials.

## Results

### The baseline characteristics of the study population

Table 1 presents the characteristics of 469 participants recruited and enrolled in the study. Most participants (70%) were aged 15–64. The average age was 53±18 years, with an age range of 16–90 years. Females made up the majority (58%). The majority of participants were Caucasian/white (75%), while 70% identified as religious. Most respondents were employed (54%), and a significant proportion were based in Leicester (32%). A considerable number of patients (85%) spoke English as their first language, 35% reported lower-limb MSK health issues and the majority (52%) had no history of chronic illness within the last 6 months prior to completing the survey. Additionally, 37% had a higher degree, professional qualifications or equivalent higher education, including those obtained abroad (table 1).

**Table 1:** Baseline demographics for the study population

Characteristic*	N=469*
Demographics Gender	
Female	272 (58%)
Male	197 (42%)
Age	
15–64 years	326 (70%)
≥65 years	139 (30%)
Ethnicity	
Caucasian/white	346 (75%)
Asian/Asian British	73 (16%)
Black/African/Caribbean/black British	27 (6%)
Mixed/multiple ethnic groups	14 (3%)
Region	
Leicester	150 (32%)
Manchester	141 (30%)
Oxford	99 (21%)
London	79 (17%)
Health and socioeconomic predictors History of past and/or present illness	
No	241 (52%)
Yes	221 (48%)
Patient chief complaint	
Lower limb problems	160 (35%)
Other musculoskeletal problems	150 (32%)
Upper limb problems	95 (21%)
Multiple musculoskeletal problems	56 (12%)
Religion	
Religious	321 (70%)
Non-religious	140 (30%)
Native English speaker	
Yes No	398 (85%) 69 (15%)
Education	
Higher degree or equivalent	170 (37%)
GCSEs† or equivalent	115 (25%)
No formal qualifications	65 (13%)
≥2 A-levels or equivalent	59 (11%)
Non-disclosed	50 (14%)
Employment status	
Working	252 (54%)
Not working	198 (43%)

* n=469.

† GCSEs General Certificate of Secondary Education.

### Awareness of biobanks, knowledge of the use of biological samples and their storage after sample collection

The study population’s awareness of biobanks showed that 63% broadly expressed no prior knowledge of the term ‘biobank’. Among them, just over half of the participants (51%) stated they were not interested in learning more about biobanks, while 49% expressed interest in learning more. Over half of the participants (53%) understood the terms ‘biological sample’ and ‘bio-specimen’ (table 2).

**Table 2:** Participants’ awareness and knowledge of biobanks and willingness to donate

Constructs*	Yes	Not sure	No
Participants’ knowledge of sample use			
Knowledge on the use of blood samples for medical research	402 (86)	14 (3)	51 (11)
Knowledge on the use of tissue samples for medical research	379 (81)	23 (5)	64 (14)
Knowledge on the use of urine samples for medical research	354 (76)	25 (5)	88 (19)
Knowledge on the use of saliva samples for medical research	346 (74)	25 (5)	96 (21)
Total ‘Knowledge of Sample Use’ score (mean±SD)	10.53±2.46		
Participant knowledge of surgical waste tissue value and management			
Knowledge on patient health data being valuable for medical research	332 (71)	52 (11)	81 (17)
Knowledge on the disposal of surgical waste tissue	286 (61)	35 (8)	144 (31)
Knowledge on the value of surgical waste tissue in medical research	205 (44)	56 (12)	205 (44)
Knowledge on storage of surgical waste tissue samples for future research	242 (52)	62 (13)	162 (35)
Total ‘Knowledge’ score (mean±SD)	6.47±2.30		
Participants’ willingness to consent to use of clinical information and biological samples for medical research			
Clinical information	309 (66)	85 (19)	71 (15)
Blood sample	339 (73)	43 (10)	82 (17)
Saliva sample	359 (77)	38 (8)	68 (15)
Urine sample	362 (79)	30 (6)	69 (15)
Surgical waste tissue sample	354 (77)	49 (11)	54 (12)
Healthy tissue	244 (53)	87 (19)	127 (28)
Total ‘Willingness’ score (mean±SD)	15.2±3.71		
Influence of key biobanking terminology on willingness to donate			
Awareness of the term ‘biobanks’	157 (37)	-	269 (63)
Knowledge of the terms ‘biological sample’ and ‘biospecimen’	250 (53)	106 (23)	109 (24)

* n(%)

On average, participants reported a high level of knowledge regarding the use of HBSs (10.53±2.46 out of 12) and surgical waste tissue management for biomedical research (6.47±2.30 out of 9). The results also showed varying levels of knowledge regarding the use of different biological samples for medical research, with a significant percentage (86%) aware of the use of blood samples. In comparison, similar percentages showed awareness of tissue (81%), urine (76%) and saliva (74%) samples. Participants demonstrated moderate knowledge (61%) of surgical waste tissue disposal. However, only 44% recognised the value of surgical waste tissue for medical research. About half the participants (52%) were aware that surgical tissue samples could be stored for future research use (see table 2).

Of all the socio-demographic variables included in the ordinal logistic model, only educational qualification was statistically significant for the knowledge of sample use for biomedical research (table 3). Participants with no formal education had 70% lower odds of reporting higher knowledge than those with higher degrees (OR 0.30, 95% CI 0.14 to 0.61; p<0.001). No other level of educational qualification was significantly associated with knowledge of use of HBS for biomedical research. No statistically significant associations were found for age group, gender, ethnicity, religion, employment status, region or a history of past and/or present illness. Full results for all predictors are detailed in table 3.

**Table 3:** Factors associated with knowledge of the sample use for medical research and knowledge of surgical waste tissue value and management

Characteristic*	Sample use		Surgical waste tissue	Management
	OR (95% CI)	P values	OR (95% CI)	P values
Age group				
Elderly (≥65 years)	1.22 (0.63 to 2.36)	0.56	1.33 (0.81 to 2.21)	0.26
Gender				
Male	1.17 (0.72 to 1.90)	0.53	0.70 (0.48 to 1.01)	0.059
Ethnicity				
Asian/Asian British	0.82 (0.40 to 1.68)	0.59	0.51 (0.29 to 0.88)	0.016
Black/African/Caribbean/black British	1.07 (0.36 to 3.14)	0.91	0.53 (0.24 to 1.18)	0.12
Mixed/multiple ethnic groups	0.76 (0.20 to 2.95)	0.70	0.53 (0.19 to 1.47)	0.22
Education				
No formal qualifications	0.30 (0.14 to 0.61)	0.001	0.29 (0.16 to 0.52)	<0.001
≥2 A-levels or equivalent	0.59 (0.28 to 1.28)	0.18	0.44 (0.24 to 0.79)	0.006
GCSEs or equivalent	0.92 (0.47 to 1.83)	0.82	0.61 (0.37 to 1.01)	0.055
Prefer not to say	0.61 (0.27 to 1.38)	0.24	0.95 (0.49 to 1.85)	0.89
Employment status				
Not working	0.76 (0.40 to 1.42)	0.39	0.96 (0.60 to 1.53)	0.85
Religion				
Non-religious	1.29 (0.72 to 2.31)	0.39	1.07 (0.69 to 1.67)	0.76
Region				
London	0.91 (0.42 to 1.98)	0.82	1.20 (0.68 to 2.12)	0.53
Manchester	0.62 (0.34 to 1.14)	0.12	1.12 (0.70 to 1.81)	0.63
Oxford	1.08 (0.51 to 2.29)	0.83	1.23 (0.71 to 2.14)	0.47
HPP				
Yes	1.21 (0.73 to 2.00)	0.46	1.37 (0.93 to 2.01)	0.11

Note: Estimates are from multivariate ordinal logistic regression (reference category=high). *Reference groups: age <65 years; female; Caucasian/white ethnicity; university-level or higher education; employed; religious; Leicester; no HPP. *Model omnibus test=(sample use (χ²(15)=12.21, p=0.66); surgical waste tissue value and management (χ²(15)=16.52, p=0.35).

GCSE, General Certificate of Secondary Education; HPP, History of Past and/or Present Illness.

The analysis of knowledge of surgical waste tissue use and management for biomedical research identified both educational qualification and ethnicity as significant predictors (table 3). Participants of Asian/Asian British ethnicity were 49% less likely to report higher knowledge compared with Caucasian/white participants (OR=0.51, 95% CI 0.29 to 0.88, p=0.016). Other ethnic groups were not significantly associated with this knowledge outcome. Compared with those holding higher degrees, participants with ≥2 A-levels or equivalent were 56% less likely to report higher knowledge (OR=0.44, 95% CI 0.24 to 0.79, p=0.006); those with no formal education were 71% less likely (OR=0.29, 95% CI 0.16 to 0.52, p<0.001); participants with General Certificate of Secondary Education or equivalent were 39% less likely (OR=0.61, 95% CI 0.37 to 1.01, p=0.055), although this did not reach statistical significance. Other variables, including age group, gender, religion, employment status, history of past and/or present illness and region, were not significant predictors.

### Participants’ willingness to consent to clinical information, biological samples and biospecimens for biomedical research

Table 2 summarises participants’ willingness to engage in biobanking initiatives. The total score for the willingness construct was 15.2±3.71 out of 18, indicating that most participants displayed a high level of readiness to consent to participate, with some variance in responses depending on the type of biological sample. Most respondents demonstrated a positive attitude towards donating their biological samples: 73% were willing to consent to blood samples, 77% to saliva samples, 79% to urine samples and 77% to surgical waste tissue samples. Participants (66%) agreed to the use of their clinical information for medical research. Among healthy tissue samples, about half of the participants (53%) were willing to donate healthy tissue for research.

The ordinal logistic model confirmed significant associations for ethnicity, education and region. Results revealed that participants identifying as Asian/Asian British were 71% less likely to report a higher willingness than Caucasian/white participants (OR=0.29, 95% CI 0.15 to 0.56, p<0.001). Those identifying as black/African/Caribbean/black British were 77% less likely to donate compared with Caucasian/white participants (OR=0.23, 95% CI 0.08 to 0.61, p=0.004). Relative to those with university/higher degrees, individuals with no formal education were 60% less likely to report greater willingness (OR=0.40, 95% CI 0.18 to 0.91, p=0.029), while those who preferred not to disclose their education level were 73% less likely (OR=0.27, 95% CI 0.13 to 0.57, p<0.001). Regional differences were also observed. Participants from Manchester were more than two times as likely to report a higher willingness to donate HBSs than those in Leicester (OR=2.18, 95% CI 1.09 to 4.33, p=0.027). Age, gender, religion, employment status, history of past and/or present illness and residence in London or Oxford were not significantly associated with willingness to donate (table 4).

**Table 4:** Factors associated with willingness to donate for biomedical research

Characteristic*	Willingness to donate	
	OR (95% CI)	P values
Age group		
Elderly (≥65 years)	1.42 (0.70 to 2.88)	0.33
Gender		
Male	0.81 (0.49 to 1.33)	0.41
Ethnicity		
Asian/Asian British	0.29 (0.15 to 0.56)	<0.001
Black/African/Caribbean/black British	0.23 (0.08 to 0.61)	0.004
Mixed/multiple ethnic groups	1.26 (0.26 to 6.00)	0.77
Education		
No formal qualifications	0.40 (0.18 to 0.91)	0.029
≥2 A-levels or equivalent	0.57 (0.24 to 1.33)	0.19
GCSEs or equivalent	0.91 (0.45 to 1.85)	0.80
Prefer not to say	0.27 (0.13 to 0.57)	<0.001
Employment status		
Not working	1.06 (0.55 to 2.02)	0.87
Religion (ref:)		
Non-religious	0.97 (0.52 to 1.81)	0.92
Region		
London	0.65 (0.34 to 1.26)	0.20
Manchester	2.18 (1.09 to 4.33)	0.027
Oxford	1.41 (0.64 to 3.13)	0.40
HPP		
Yes	1.51 (0.89 to 2.56)	0.13

Note: Estimates are from multivariate ordinal logistic regression (reference category=high). *Reference groups: age <65 years; female; Caucasian ethnicity; university-level or higher education; employed; religious; Leicester; no HPP. *Model omnibus test=(χ²(15)=12.21, p=0.66).

GCSEs, General Certificate of Secondary Educations; HPP, History of Past and/or Present Illness.

The analysis of how key terminology related to biobanking influences willingness was also conducted. Awareness of the term ‘biobank’ was significantly linked to a greater willingness to donate. Participants who knew the term had more than two times the odds of higher willingness compared with those who did not (OR=2.16, 95% CI 1.23 to 3.77, p=0.007). In contrast, familiarity with other terms such as ‘biological sample’ and ‘biospecimen’ was not statistically significant (table 5).

**Table 5:** The influence of awareness of the term ‘biobanks’, knowledge of the terms ‘biological sample’ and ‘biospecimen’ on willingness to donate

Predictor	Willingness to donate	
	OR (95% CI)	P values
Knowledge of the terms ‘biological sample’ and ‘biospecimen’	1.25 (0.95 to 1.65)	0.110
Awareness of the term ‘biobanks’	2.16 (1.23 to 3.77)	0.007

Note: Estimates are from ordinal logistic regression (reference category=high).

### Factors that affect participant willingness and hesitancy to take part in biobanking initiatives

**Figure 1:**
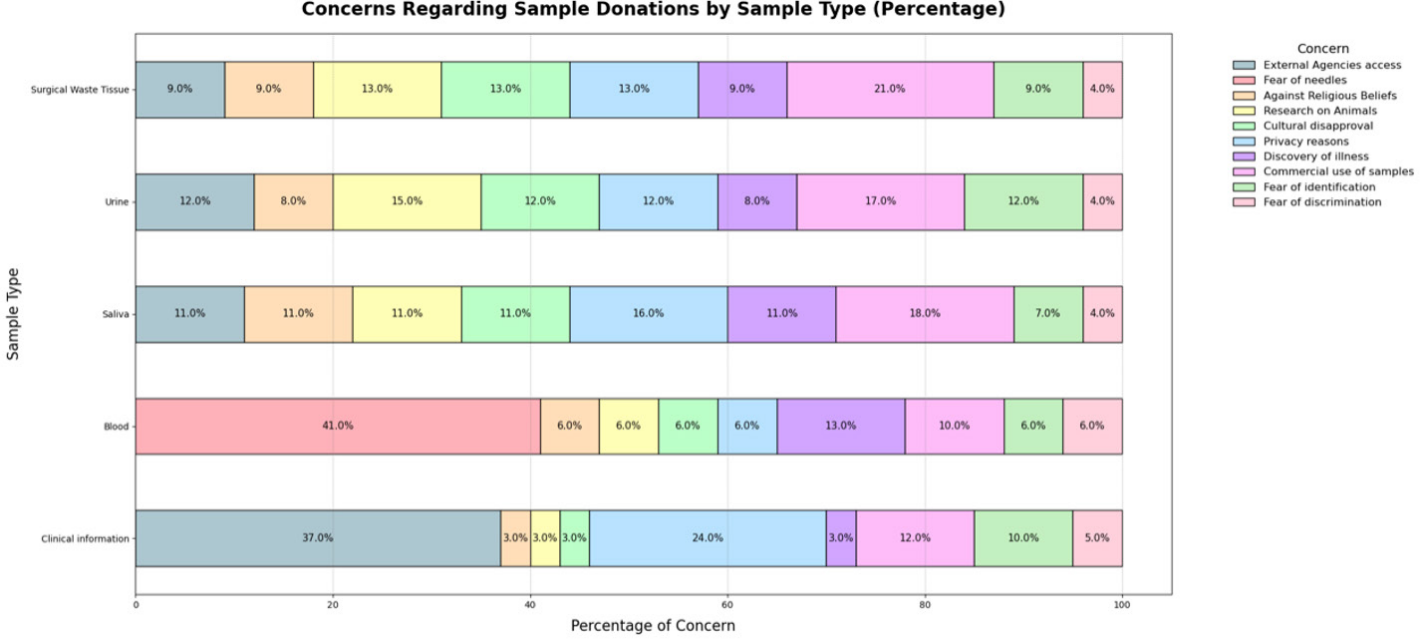
Concerns regarding sample donations by sample type (%). Participants most commonly cited concerns about sample commercialisation (for surgical waste, 21%, urine 17% and saliva, 18%), fear of needles (for blood samples, 41%), data sharing with external agencies (for clinical information) and the need for full context or risks involved (for healthy tissue). Patterns of objection and hesitation were consistent across samples, with informed consent noted as essential even among willing donors.

Figure 1 summarises the reasons participants provided for their hesitation or unwillingness to donate. Participants’ objections (no) and hesitations (not sure) showed a similar pattern across the samples. For surgical waste tissue (21%), urine (17%) and saliva (18%), participants mainly expressed concerns about the commercialisation of samples. For blood samples (41%), the main reasons cited by those who objected or hesitated were fear of needles and the potential discovery of illness (31%). Participants also raised concerns about their clinical information being used by other agencies, such as the police, social services and local councils (37%). Regarding healthy tissue donation, participants often noted they needed the full context behind the sample request to make an informed decision. In contrast, others highlighted risks associated with obtaining healthy tissue during routine surgery. Even when participants agreed to donate, they emphasised that informed consent would need to be obtained before they fully agreed.

## Discussion

Biobanks form the infrastructural foundation for the global adoption of genomic technologies that will facilitate the transition of healthcare approaches to precision medicine.^27^,^29^ This study reveals a key paradox in the MSK patient cohort across four sites in England: while awareness of the term ‘biobank’ was low (only 37% were familiar), participants demonstrated high knowledge of the use and value of HBSs in biomedical research. This pattern mirrors findings from the UK,^17^ where 87% of the public view biobanks as important, with similar findings for a Scottish-focused survey (82%),^30^ despite limited awareness of the terminology. Our awareness finding also sits at the lower end of global patterns, ranging from 28% to 68% across countries such as Latvia,^31^ Jordan,^32^ Morocco,^33^ China^34^ and the USA.^35^

It was therefore important to examine awareness of the term ‘biobank’ alongside familiarity with related terminology such as ‘biological sample’ and ‘biospecimen’, especially considering the two decades of widespread public campaigns by the UK Biobank. Awareness of the term ‘biobank’ may indicate recognition of formal, institutional research infrastructures, which can foster trust. In contrast, familiarity with specific sample-related terminology might require deeper engagement or prior exposure to clinical research settings. This distinction may explain why awareness of the term, rather than technical familiarity with samples, was associated with higher willingness to donate: the term ‘biobank’ may act as a gateway to perceived legitimacy. Furthermore, the lack of interest in learning more about biobanks among half of the unaware participants of the terminology suggests that barriers to participation may be rooted in deeper issues of mistrust or disengagement rather than a mere lack of information and warrant further qualitative exploration.^36^

The varied knowledge levels across HBS types in our findings align with a 2000 Wellcome Trust report, which concluded that UK public knowledge of HBS use varies by sample type. Even with high levels of knowledge, specific knowledge gaps were evident, particularly regarding the research use of surgical waste tissue. This finding offers promising insights into the barriers to constructing single-cell MSK reference atlases, as most of the tissues requested for biobanking are surgical waste tissue.^37^

High willingness to donate HBSs, especially surgical waste, saliva and urine was observed, though this willingness varied by socio-demographic factors. Interestingly, the pronounced regional difference between Manchester and other locations in England suggests attitudes towards donation are not absolute among MSK patients. Our results also reinforce the well-documented influence of higher education on greater understanding of various aspects of biobanking and willingness to donate.^38 39^ Where our study adds another layer of detail is in confirming that within an MSK patient cohort, significant ethnic disparities in knowledge and willingness to donate persist.^12 40^ Lower willingness to donate among Asian and Black participants directly challenges the efforts to build ancestrally diverse MSK atlases with biological samples that are representative of the population. Prior studies have attributed these findings to historical mistrust, socio-cultural sensitivities and perceived lack of community engagement, which often challenge efforts to improve inclusion.^39 41^ Lack of engagement of ethnic minorities in biobanking initiatives has real-world implications, especially for the future of precision medicine.^42^,^45^ This risks exacerbating health inequalities, as research findings may not be generalisable to the entire population. For instance, the predominance of European ancestry in genomic studies further widens this disparity, potentially contributing to reduced awareness, familiarity and perceived relevance of biobanks among these groups.^46 47^

The reasons identified in our study for reluctance to donate were multifaceted, reflecting a complex landscape of sample-specific fears (eg, needles), ethical concerns (such as the commercialisation of samples) and fundamental mistrust, particularly regarding data security and potential access by non-medical agencies. These barriers are frequently noted in the literature.^48 49^ Additional concerns, such as fears of illness detection and discrimination based on findings, reveal that a ‘one-size-fits-all’ approach to addressing the reluctance among MSK patients is unlikely to succeed. Instead, these concerns highlight well-known, persistent concerns and underscore the importance of transparent policies and robust data governance to maintain trust and support informed consent processes.^1349^,^51^ These findings also suggest that a unidirectional, information-based approach to patient education is insufficient. While awareness gaps exist, simply providing facts may not consistently alter attitudes or behaviours of patients. To address these gaps for under-represented groups, biobanking campaign resources and patient research recruitment processes must be viewed as a crucial dialogue to address these concerns, as they are primary opportunities to build trust by transparently addressing data governance, assuring of no commercial access where applicable and tailoring the conversation to specific socio-cultural contexts.

The primary strength of this study lies in its focus on a specific and relevant patient population, such as individuals with MSK conditions, who are key stakeholders in the development of ancestrally diverse MSK reference atlases. Similarly, this study offers deeper insights into multiple socio-demographic factors, identifying key predictors of knowledge regarding HBS use and surgical waste tissue management, as well as willingness to donate. However, the study has limitations. Its cross-sectional design means we can only report associations, not causality. The reliance on self-reported data may be affected by social desirability bias. Finally, although we identified significant educational, regional and ethnic differences in willingness, our quantitative approach does not fully explore the underlying reasons for this variation, indicating a need for further research. The generalisability of these findings is limited by the use of convenience sampling and the exclusion of patients who do not speak English, which may restrict the application of results to the wider UK population or those with limited literacy. Furthermore, the sample was slightly more educated than the national average. However, given that the primary target of biobanking initiatives is patients already engaged in clinical care, this cohort serves as a suitable proxy for the population most likely to be approached for donation. Therefore, despite these limitations, the findings offer valuable evidence for designing recruitment strategies within hospital-based MSK services.

This study also raises several questions for future investigation. The reasons behind participants in Manchester’s higher willingness to donate need to be explored, likely through qualitative methods, to understand which factors contribute to this positive outlier. Further research must also move beyond quantifying disparities to co-design culturally sensitive, dialogue-based engagement strategies that can effectively build trust with historically underserved communities. A future qualitative study employing a stratified sampling approach to target the specific ethnic and educational groups identified in our analysis would be a vital next step. Such work is essential to developing evidence-based resources that can bridge knowledge gaps, address deep-rooted concerns and ultimately ensure that the construction of foundational research resources, such as single-cell MSK reference atlases, is an equitable and inclusive process.

## Conclusion

This study adds to the wealth of literature on biobanking research by being the first study to explore the willingness to donate HBSs among MSK patients in England. Its findings indicate that educational, regional and ethnic gaps across diverse socio-demographic groups need to be addressed to improve patients’ familiarity with the concept and practice of biobanking and to successfully develop ancestrally inclusive biomedical research. Future efforts must prioritise culturally sensitive engagement to enhance understanding and build trust, ensuring equitable and diverse participation and a tailored approach to engage a diverse pool of potential donors effectively.

## Supplementary material

10.1136/bmjopen-2025-111653 online supplemental file 1

## Data Availability

Data are available in a public, open access repository.
