# Supplementary material for: Biobanking knowledge and donation willingness among musculoskeletal patients in England: a multisite cross-sectional study
Source: BMJ Open. 2026 Feb 15;16(2):e111653. doi: 10.1136/bmjopen-2025-111653 (PMC12911750; doi:10.1136/bmjopen-2025-111653)
Supplement: online supplemental file 1 [file bmjopen-16-2-s001.pdf]

Supplementary Table 1: Patterns of missing data across survey items in the MISSION Survey

Variable	Number Missing	Percent Missing
**Participant ID**	0	0
**Age**	4	0.008528785
**Gender**	0	0
**Ethnicity**	0	0
**Religion**	3	0.006396588
**Region**	0	0
**First Language Spoken**	2	0.004264392
**Employment Status**	3	0.006396588
**Type of Employment**	196	0.4179104
**Postcode**	11	0.02345416
**Education**	10	0.02132196
**History of present or past illness**	7	0.01492537
**Chief Complaint**	8	0.01705757
**Q12**	4	0.008528785
**Q13**	2	0.004264392
**Q14**	3	0.006396588
**Q15**	2	0.004264392
**Q16**	2	0.004264392
**Q17**	4	0.008528785
**Q18**	3	0.006396588
**Q19**	3	0.006396588
**Q20**	4	0.008528785
**Q21**	4	0.008528785
**Q22**	5	0.01066098
**Q23**	4	0.008528785
**Q24**	8	0.01705757
**Q25**	12	0.02558635
**Q26**	11	0.02345416
**Q27**	43	0.09168443

Supplementary Table 2: Results of Little's MCAR Test.

Statistic	Value
Chi-Square ($\chi 2$)	1460.73
Degrees of Freedom (df)	1156
P-value (p)	$< .001$

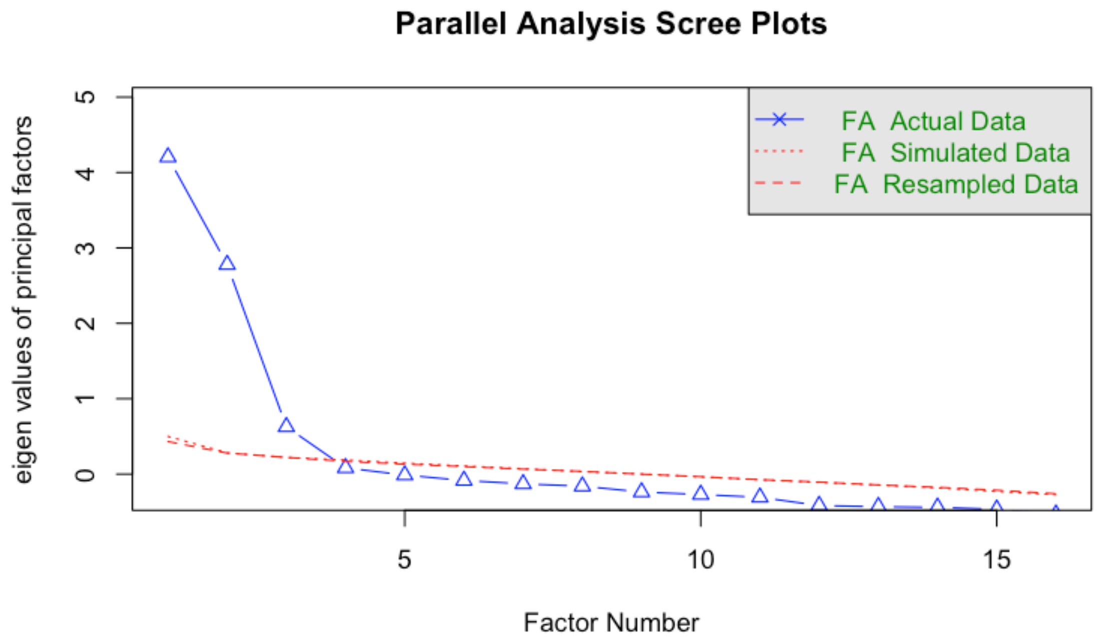

Supplementary Figure 1. Parallel Analysis Scree Plot of factor loadings. The figure indicates 3 three stable and clear factor loadings explained by survey items.

Supplementary Table 3: Exploratory factor analysis of the MISSION Survey items assessing willingness to donate and knowledge of biobanking.

Item	Factor 1 (Willingness to donate)	Factor 2 (Knowledge of Sample Use)	Factor 3 (Knowledge of Surgical Waste Tissue use and management)
Q23	0.943		
Q24	0.903		
Q22	0.834		
Q25	0.770		
Q21	0.665		
Q26	0.550		
Q15		0.837	
Q13		0.812	
Q16		0.786	
Q14		0.575	
Q19			0.764
Q18			0.76
Q17			0.664
Pooled Cronbach's alpha	0.90	0.85	0.78

Supplementary Table 4: Exploratory factor analysis of the MISSION Survey items (no imputation) assessing willingness to donate and knowledge of biobanking [Before poor factor loading removal].

Item	F1 (Willingness to donate)	F2 (Knowledge of Sample Use)	F3 (Knowledge of Surgical Waste Tissue Use and Management)
Q12		0.265	0.308
Q13		0.807	
Q14		0.578	0.234
Q15		0.837	
Q16		0.788	
Q17	0.107		0.662
Q18			0.768
Q19			0.771
Q20		0.217	0.383
Q21	0.66		
Q22	0.832		
Q23	0.946		
Q24	0.901		
Q25	0.761		
Q26	0.544		0.126
Q27	0.104	0.103	0.334

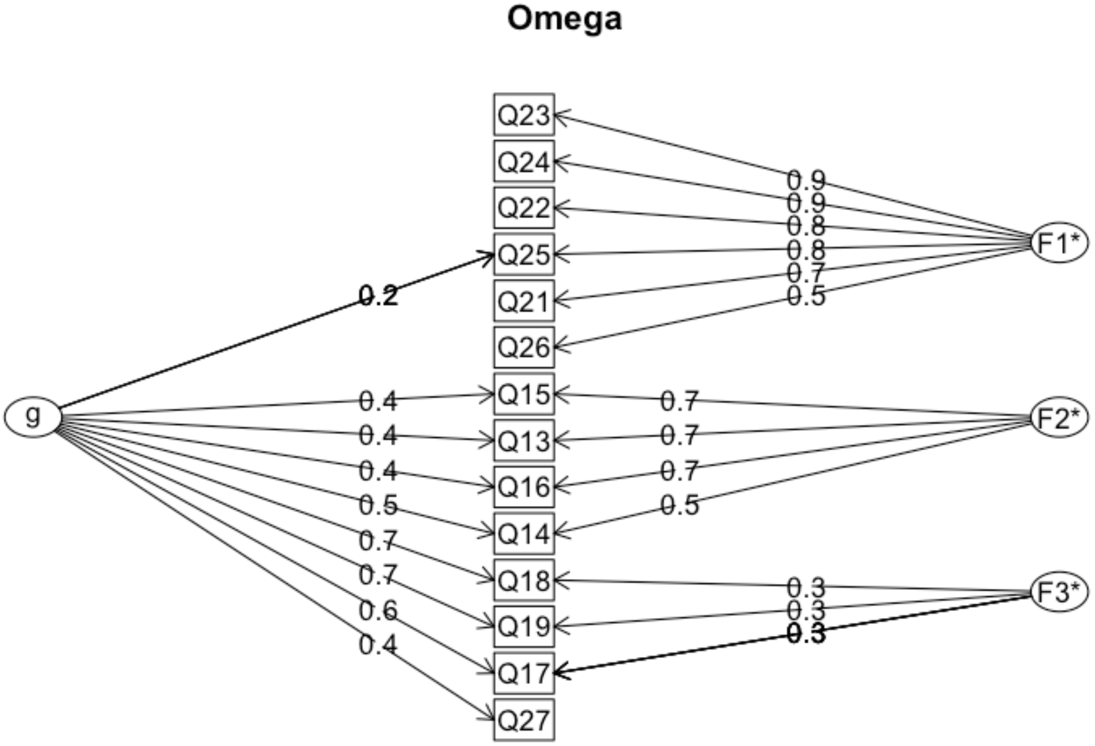

Supplementary Figure 2. Bifactor omega model showing standardized loadings for a three-factor solution. All items load on a general factor (g) and on one of three specific factors (F1–F3), indicating three stable item clusters in addition to a general dimension

Supplementary Table 5: Psychometric Properties of Outcome Measures

Outcome	Number of items	M (SD)	Skew	Alpha coefficient	Omega coefficient
Willingness to Donate	6	15.2/ 18 (3.47)	-1.68	0.90 (95% CI: 0.88-0.92)	0.91
Knowledge of Sample Use	4	10.5 / 12 (2.46)	-1.38	0.85 (95% CI: 0.81-0.89)	0.86
Knowledge of Surgical Waste Tissue Use and Management	3	6.47 / 9 (2.30)	-1.69	0.78 (95% CI: 0.74-0.82)	0.75
