# Supplementary material for: Biobanking knowledge and donation willingness among musculoskeletal patients in England: a multisite cross-sectional study
Source: BMJ Open. 2026 Feb 15;16(2):e111653. doi: 10.1136/bmjopen-2025-111653 (PMC12911750; doi:10.1136/bmjopen-2025-111653)
Supplement: Supplementary file 2 [file bmjopen-2025-111653.reviewer_comments.pdf]

PEER REVIEW HISTORY

BMJ Open publishes all reviews undertaken for accepted manuscripts. Reviewers are asked to complete a checklist review form and are provided with free text boxes to elaborate on their assessment. These free text comments are reproduced below.

ARTICLE DETAILS

Title (Provisional)

Biobanking Knowledge and Donation Willingness Among Musculoskeletal Patients in England: A Multi-site Cross-sectional Study

Authors

Boakye Serebour, Tracy; Kerasidou, Angeliki; Gray , Naomi; Griffin, Xavier Luke; Peach, Chris; Singh, Harvinder pal; Wheway , Kim; Ambrosio , Maria da Graca; Baldwin, Mathew; Snelling, Sarah Jane Bothwell

---

VERSION 1 - REVIEW

Reviewer **1**

Name **Lin, Sien**

Affiliation **The University of Hong Kong, Department of Orthopaedics and Traumatology**

Date **25-Oct-2025**

COI **None**

This is a well-conducted cross-sectional study that addresses the attitudes of musculoskeletal patients in England towards biobanking and tissue donation. The manuscript is clearly written, the methodology is sound, and the statistical analysis is appropriate. Here are my suggestions:

1. While the authors compared demographics to census data, the sample may not perfectly representative. The "No formal qualifications" group (13%) looks smaller than the national average, and the sample is heavily weighted towards Leicester and Manchester (62% combined). This could affect the generalizability of the regional finding.

2. Regarding the language, it seems the majorities speak English. There could be bias in interpreting the willingness in the the non-English minority.

3. It seems only one figure in the maintext, however, figure 2 was described in page 15.

Reviewer **2**

Name **Talarico, Rosaria**

**Affiliation**	**University of Pisa, Rheumatology Unit**
**Date**	**28-Oct-2025**
**COI**	**None**

This is a well-conceived and timely study exploring knowledge and willingness to donate biological samples among musculoskeletal (MSK) patients across multiple UK sites. The topic is of clear relevance to biomedical research, particularly in the context of building ancestrally inclusive biobanks and enhancing public engagement with genomic medicine. The manuscript is well written, the methodology is generally appropriate, and the findings are clearly presented and discussed in light of existing literature. The study provides valuable insights into sociodemographic disparities affecting participation in biobanking and offers practical implications for improving patient engagement.

Strengths

The study addresses a relevant and underexplored area — biobanking attitudes among MSK patients — with important implications for equity and inclusion in biomedical research.

The multicentre design, covering diverse UK regions, increases the representativeness of the findings.

Limitation

As the authors stated, the study would have benefited from a mixed-method approach that included quantitative and qualitative data collection, which would have provided more insight into understanding

participants' unwillingness to donate HBSs. I would suggest encouraging this approach in the future.

Minor comments:

- The use of opportunistic (convenience) sampling is acknowledged, but its implications for generalizability should be discussed more explicitly, particularly regarding potential selection bias at recruitment sites.

- Ensure that all abbreviations (HBS, PPI, EFA, MICE) are defined at first mention in both the Abstract and the main text.

VERSION 1 - AUTHOR RESPONSE

<u>Response to Reviewer 1</u>

Comment 1: The "No formal qualifications" group (13%) looks smaller than the national average... This could affect the generalizability.

Response: We acknowledge the sample is slightly more educated than the national average

(approx. 18% have no qualifications vs 13% in our sample). However, our target population was specifically MSK patients in clinical settings, who are the primary target for biobanking recruitment. Change in Manuscript (Page 16): We have updated the Limitations section to acknowledge that the sample was "slightly more educated than the national average" and that this limits generalizability to the wider public.

Comment 2: Regarding the language... There could be bias in interpreting the willingness in the non-English minority.

Response: We agree. The exclusion of non-English speakers is a limitation.

Change in Manuscript (Page 16): We have added to the Limitations: "The generalisability of these findings is limited by... the exclusion of patients who do not speak English, which may restrict the application of results to the wider UK population."

Comment 3: It seems only one figure in the main text, however, figure 2 was described in page 15.

Response: The reference to Figure 2 was a typographical error.

Change in Manuscript (Page 15): We have removed the incorrect reference to "Figure 2" in the Results section regarding Omega coefficients.

Response to Reviewer 2

Comment 1: The study would have benefited from a mixed-method approach.

Response: We agree and have highlighted this in our future research plans.

Change in Manuscript (Page 17): "A future qualitative study employing a stratified sampling approach... would be a vital next step."

Comment 2: The use of opportunistic (convenience) sampling... implications for generalizability should be discussed.

Response: We have discussed this in the Limitations section.

Change in Manuscript (Page 16)

Comment 3: Ensure that all abbreviations (HBS, PPI, EFA, MICE) are defined.

Response: We have verified definitions in the text.

VERSION 2 - REVIEW

**Reviewer**	**1**
**Name**	**Lin, Sien**
**Affiliation**	**The University of Hong Kong, Department of Orthopaedics and Traumatology**
**Date**	**08-Dec-2025**
**COI**	

The authors have successfully addressed my concerns.

**Reviewer**	**2**
**Name**	**Talarico, Rosaria**
**Affiliation**	**University of Pisa, Rheumatology Unit**
**Date**	**18-Dec-2025**
**COI**	

This manuscript presents a well-designed multi-site cross-sectional study exploring knowledge of biobanking and willingness to donate biological samples among musculoskeletal (MSK) patients in England. The topic is timely and highly relevant, particularly in light of ongoing efforts to build ancestrally inclusive biobanks and single-cell atlases. The study benefits from a relatively large sample size, recruitment across four NHS sites, and a rigorous statistical approach, including ordinal regression models and multiple imputation for missing data.

Overall, the manuscript is clearly written, methodologically sound, and aligns well with the scope of BMJ Open. The findings add incremental but meaningful evidence to the literature on patient engagement in biobanking, especially within a specific clinical population. I believe the paper is suitable for publication pending minor revisions, primarily aimed at improving clarity and interpretability rather than addressing fundamental methodological concerns.

- first of all, while the authors appropriately acknowledge the use of opportunistic/convenience sampling, it would be helpful to briefly clarify in the Methods section how this might have influenced participant characteristics (e.g. higher engagement, greater baseline trust in healthcare settings), beyond what is already discussed in the limitations.

- if I have correctly understood, the distinction between awareness of the term “biobank” and broader knowledge of sample use is interesting and well-motivated. The Discussion could benefit from a slightly clearer conceptual framing of these constructs, particularly

when interpreting why awareness—but not familiarity with other terminology—was associated with willingness to donate.

- while the transformation of summed scores into ordinal categories ("Low", "Moderate", "High") is reasonable, providing a brief justification for how the cut-off values were determined (for example, using distribution-based thresholds) would enhance methodological transparency and facilitate reproducibility.

VERSION 2 - AUTHOR RESPONSE

Reviewer: 2
Dr. Rosaria Talarico, University of Pisa
Comments to the Author:
This manuscript presents a well-designed multi-site cross-sectional study exploring knowledge of biobanking and willingness to donate biological samples among musculoskeletal (MSK) patients in England. The topic is timely and highly relevant, particularly in light of ongoing efforts to build ancestrally inclusive biobanks and single-cell atlases. The study benefits from a relatively large sample size, recruitment across four NHS sites, and a rigorous statistical approach, including ordinal regression models and multiple imputation for missing data.

Overall, the manuscript is clearly written, methodologically sound, and aligns well with the scope of BMJ Open. The findings add incremental but meaningful evidence to the literature on patient engagement in biobanking, especially within a specific clinical population. I believe the paper is suitable for publication pending minor revisions, primarily aimed at improving clarity and interpretability rather than addressing fundamental methodological concerns.

- first of all, while the authors appropriately acknowledge the use of opportunistic/convenience sampling, it would be helpful to briefly clarify in the Methods section how this might have influenced participant characteristics (e.g. higher engagement, greater baseline trust in healthcare settings), beyond what is already discussed in the limitations.
- if I have correctly understood, the distinction between awareness of the term "biobank" and broader knowledge of sample use is interesting and well-motivated. The Discussion could benefit from a slightly clearer conceptual framing of these constructs, particularly when interpreting why awareness—but not familiarity with other terminology—was associated with willingness to donate.
- while the transformation of summed scores into ordinal categories ("Low", "Moderate", "High") is reasonable, providing a brief justification for how the cut-off values were determined (for example, using distribution-based thresholds) would enhance methodological transparency and facilitate reproducibility.
